# Stereodynamic tetrahydrobiisoindole “NU-BIPHEP(O)”s: functionalization, rotational barriers and non-covalent interactions

**DOI:** 10.3762/bjoc.12.141

**Published:** 2016-07-14

**Authors:** Golo Storch, Sebastian Pallmann, Frank Rominger, Oliver Trapp

**Affiliations:** 1 Organisch-Chemisches Institut, Ruprecht-Karls Universität Heidelberg, Im Neuenheimer Feld 270, 69120 Heidelberg, Germany

**Keywords:** atropisomer, enantioselective DHPLC, ligand design, non-covalent interactions, Okamoto phases, phosphine ligand, stereodynamic ligands

## Abstract

Stereodynamic ligands offer intriguing possibilities in enantioselective catalysis. “NU-BIPHEPs” are a class of stereodynamic diphosphine ligands which are easily accessible via rhodium-catalyzed double [2 + 2 + 2] cycloadditions. This study explores the preparation of differently functionalized “NU-BIPHEP(O)” compounds, the characterization of non-covalent adduct formation and the quantification of enantiomerization barriers. In order to explore the possibilities of functionalization, we studied modifications of the ligand backbone, e.g., with 3,5-dichlorobenzoyl chloride. Diastereomeric adducts with Okamoto-type cellulose derivatives and on-column deracemization were realized on the basis of non-covalent interactions. Enantioselective dynamic HPLC (DHPLC) allowed for the determination of rotational barriers of Δ*G*^‡^_298K_ = 92.2 ± 0.3 kJ mol^−1^ and 99.5 ± 0.1 kJ mol^−1^ underlining the stereodynamic properties of “NU-BIPHEPs” and “NU-BIPHEP(O)s”, respectively. These results make the preparation of tailor-made functionalized stereodynamic ligands possible and give an outline for possible applications in enantioselective catalysis.

## Introduction

Axially chiral biaryl compounds such as BINAP (2,2’-bis(diphenylphosphino)-1,1’-binaphthyl) represent widely used and highly efficient ligands that can be applied in a variety of enantioselective catalytic transformations. Unlike BINAP, the related stereodynamic BIPHEP (2,2’-bis(diphenylphosphino)-1,1’-biphenyl) ligands have a significantly lower barrier of rotation around the central C–C bond regarding the conversion of the enantiomers into one another. This enables fast enantiomerization at room temperature.

This, however, does not conflict with their usage in enantioselective catalysis. Noyori and Mikami reported the stereochemical alignment of BIPHEP ligands in ruthenium complexes upon addition of chiral diamine co-ligands [1–2]. The resulting complexes were successfully employed in enantioselective ketone hydrogenation. Further examples of such systems are BIPHEP complexes of rhodium [3–6], palladium [7–8], platinum [9–10] and gold [11–13] in combination with chiral co-ligands or counter ions that are used after alignment of the ligand’s axial chirality.

One major advantage of stereodynamic ligands is that there is no need for separate preparation of one ligand enantiomer as long as their chirality can be controlled by chiral additives or auxiliaries. In addition, the simultaneous presence of both axially chiral BIPHEP enantiomers can be beneficial as this allows bidirectional control of enantioselectivity depending on temperature [14–15]. In this approach, both product enantiomers of an enantioselective transformation can be addressed selectively by fine tuning of the conditions prior to and during catalysis.

The rotational barrier around the central C–C bond of BIPHEP ligands is a key property of stereodynamic ligands that determines the temperature required for ligand enantiomerization as well as the half-life of isolated enantiomers. The latter are of particular importance if chiral co-ligands are cleaved off prior to catalysis and if the remaining stereochemically aligned BIPHEP complex fragment serves as the active species. Therefore, detailed knowledge of the interconversion barriers of stereodynamic ligands is crucial for the choice of conditions used for stereochemical alignment and subsequent application in catalysis. A rotational barrier of 92 kJ mol^−1^ for the unsubstituted BIPHEP was determined by NMR coalescence of a partially deuterated derivative [16]. However, this method does not fulfil the requirements for a reliable rapid screening of novel stereodynamic ligands due to harsh conditions such as isotope exchange. We recently reported the rotational barriers of 3,3’ and 5,5’ substituted BIPHEP and BIPHEP(O) compounds based on enantioselective DHPLC by evaluation of elution profiles using the unified equation [17–20]. Rotational barriers were found to be between 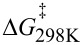 = 86.8 kJ mol^−1^ (unsubstituted BIPHEP) and 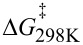 = 100.4 kJ mol^−1^. BIPHEP(O) derivatives (unsubstituted BIPHEP(O): 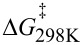 = 88.6 kJ mol^−1^) were observed to exhibit slightly increased (approximately 2 kJ mol^−1^) barriers.

Functionalization of stereodynamic BIPHEP ligands at the biaryl core offers multiple possibilities. The introduction of achiral, non-covalent interaction sites allows for ee determination of chiral analytes via NMR spectroscopy [21] as well as deracemization of the BIPHEPs with HPLC stationary phases [22].

However, introduction of functional groups which enable a modular derivatization approach is often hampered by long and tedious synthetic procedures. Doherty et al. reported a rhodium catalyzed double [2 + 2 + 2] cycloaddition strategy for a convergent synthesis of “NU-BIPHEP”s [23].

In this paper, we describe the application of Doherty’s synthetic strategy for the synthesis of stereodynamic tetrahydrobiisoindole “NU-BIPHEP(O)” compounds bearing secondary amino groups for functionalization. The attachment of a 3,5-dichlorobenzoyl binding site is reported and non-covalent interactions as well as rotational barriers are studied in solution by (D)HPLC techniques.

## Results and Discussion

### Synthesis of tetrahydrobiisoindole “NU-BIPHEP(O)s”

**Figure 1:**
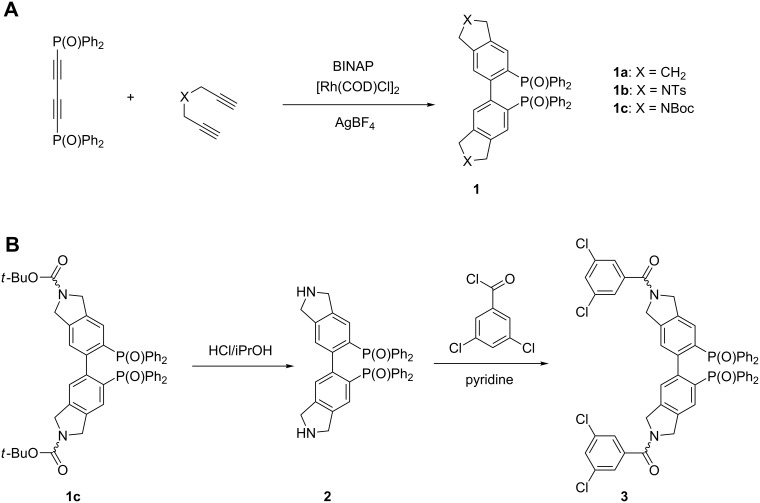
Synthetic overview of “NU-BIPHEP(O)s”. A) Rhodium catalyzed double [2 + 2 + 2] cycloaddition. B) Acidic deprotection of tetrahydrobiisoindole “NU-BIPHEP(O)” **1c** and subsequent amide bond formation with 3,5-dichlorobenzoyl chloride.

The rhodium catalyzed double [2 + 2 + 2] cycloaddition of 1,4-bis(diphenylphosphinoyl)buta-1,3-diyne and a variable diyne compound is the key step in the preparation of “NU-BIPHEPs” [23] and related biaryls [24]. Doherty et al. reported the use of various diynes yielding for instance tetrahydrobiindene **1a** and *N*-tosyl-protected tetrahydrobiisoindole **1b** as the only *N*-heterocyclic compound (Figure 1A).

Aiming at facile deprotection and enabling subsequent functionalization at the secondary amine position, we changed the strategy and used *N*-Boc dipropargylamine as the diyne compound (Figure 1A). The double cycloaddition product **1c** was obtained in 77% yield. In accordance with the report of Doherty et al., very slow addition of the diyne compound via syringe pump was crucial.

In contrast to **1a** and **1b**, three coexisting isomeric species were observed with NMR spectroscopy in CDCl_3_ for tetrahydrobiisoindole “NU-BIPHEP(O)” **1c**. This behaviour originates from an increased interconversion barrier between the *E*/*Z* isomers of the carbamate N–C(O) unit that is derived from a secondary amine. Deprotection of **1c** proceeds smoothly with 5–6 M HCl in 2-propanol and the tetrahydrobiisoindole “NU-BIPHEP(O)” **2** can be isolated as hydrogen chloride salt in 93% yield (Figure 1B).

**Figure 2:**
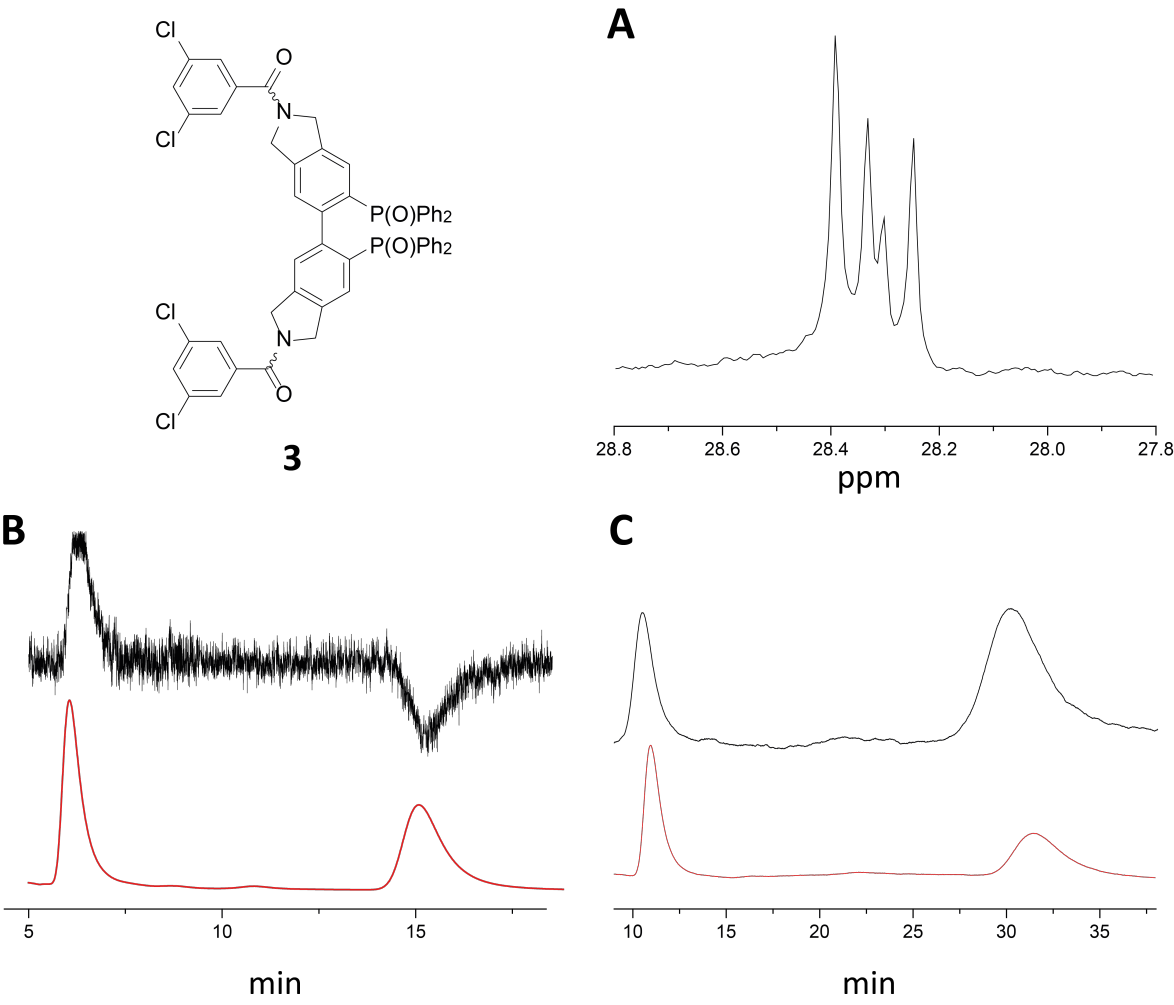
Investigation of 3,5-dichlorobenzoyl modified tetrahydrobiisoindole “NU-BIPHEP(O)” **3**. A) Three signal sets are observed in ^31^P{^1^H} NMR spectroscopy (CDCl_3_). B) HPLC–CD chromatogram (*n*-hexane/2-propanol 50:50, CHIRALPAK^®^ IA-3, 1 mL/min, 20 °C). Red: UV trace, black: CD trace. C) On-column deracemization of **3** (*n*-hexane/2-propanol 50:50, CHIRALPAK^®^ IA). Chromatogram with (black) and without (red) the analyte after being kept on the stationary phase for seven days.

Tetrahydrobiisoindole “NU-BIPHEP(O)” **2** offers various possibilities for functionalization with chiral and achiral auxiliaries or binding sites. In this study, we chose amide bond formation with 3,5-dichlorobenzoyl chloride (Figure 2B) in connection with our recent report [21] on non-covalent interaction properties of stereodynamic BIPHEP ligands with this binding site that is well known in HPLC stationary phase design. The “NU-BIPHEP(O)” **3** was isolated in 49% yield and again three isomeric species were observed by NMR spectroscopy in CDCl_3_ due to hindered rotation of the tertiary amide bond (Figure 2A).

Intriguingly, only two peaks were observed upon investigation of **3** by enantioselective HPLC applying *n*-hexane/2-propanol as mobile phase and a normal phase Okamoto-type stationary phase (CHIRALPAK^®^ IA-3). Opposing signals in HPLC–CD coupling corroborated the assumption that the peaks correspond to two axially chiral enantiomers (Figure 2B).

When left on the chiral stationary phase (*n*-hexane/2-propanol 50:50, CHIRALPAK^®^ IA) for seven days in a stopped-flow experiment, partial deracemization of **3** was observed. The final enantiomeric ratio (absolute configurations were not determined) was observed to reach approximately 72:28.

### Non-covalent interactions in solution

Non-covalent interactions are not only a key step in deracemization of stereodynamic compounds but they also allow ee determination in solution by NMR spectroscopy. However, the formation of diastereomeric adducts between chiral compounds and BIPHEP(O) [25] or BINAP(O) [26] has so far rarely been investigated. In a subsequent part of our study, we explored the non-covalent interactions of tetrahydrobiisoindole “NU-BIPHEP(O)s” in solution since they exhibit very strong interactions with Okamoto-type chiral stationary phases (CSPs) in HPLC.

Okamoto et al. reported the preparation of short CSP-analogue cellulose and amylose compounds with carbamate selector units that are soluble in organic solvents [27–30]. We prepared cellulose derivative **4** which bears 5-fluoro-2-methylphenylcarbamate binding sites [27] and investigated the formation of diastereomeric adducts with **1b** and **3** by ^31^P{^1^H} NMR spectroscopy in anhydrous CDCl_3_. Significant signal splitting was observed for both “NU-BIPHEP(O)s” due to strong non-covalent interactions. However, detailed analysis of **3** was hampered due to overlaying multiple signal sets caused by *E*/*Z* isomerism of the tertiary amide unit.

**Figure 3:**
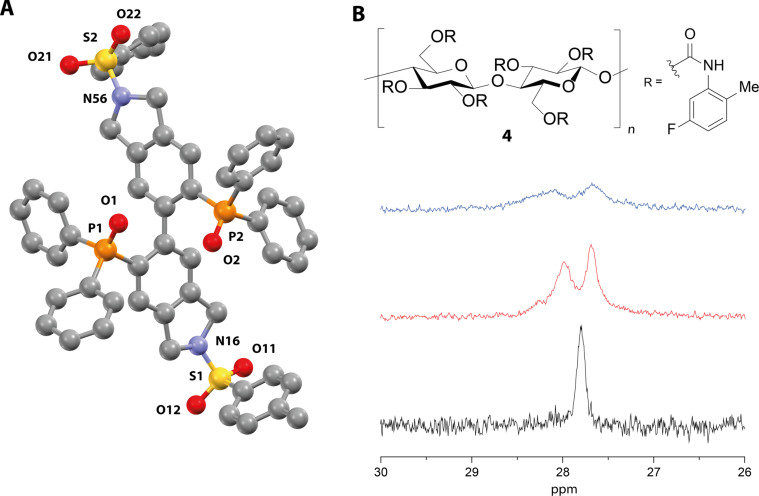
Investigation of “NU-BIPHEP(O)” **1b**. A) Solid-state structure determined by X-ray crystallography. Hydrogen atoms and methanol solvent molecules are omitted for clarity. B) Interaction studies in solution with soluble Okamoto phase **4** by ^31^P{^1^H} NMR spectroscopy. Black: spectrum of 4.7 mg (5 µmol) **1b** in 0.5 mL anhydrous CDCl_3_ (filtered through basic alumina). Red: spectrum after addition of 20 mg **4**. Blue: spectrum after addition of 0.1 mL anhydrous *n*-pentane. All NMR samples were completely dissolved.

Adding **4** to a solution of **1b** (Figure 3A, solid-state structure) in CDCl_3_ resulted in significant signal splitting of Δδ = 0.30 ppm (^31^P{^1^H} NMR). This effect could be intensified by adding *n*-pentane which increased the splitting to Δδ = 0.41 ppm although signal broadening rose simultaneously (Figure 3B).

### Determination of rotational barriers by enantioselective DHPLC

**Figure 4:**
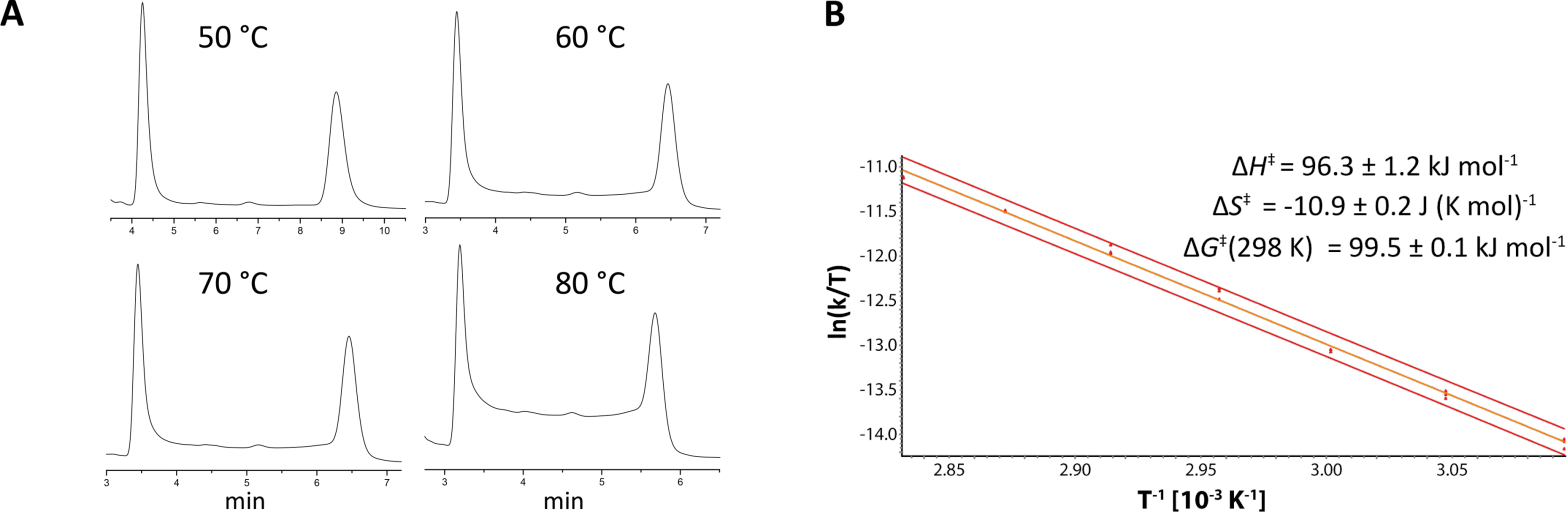
Enantioselective DHPLC investigation of tetrahydrobiisoindole “NU-BIPHEP(O)” **3**. A) Elution profiles at various temperatures with increasing plateau formation. B) Eyring plot analysis for the determination of the activation parameters Δ*H*^‡^ and Δ*S*^‡^.

To the best of our knowledge the enantiomerization properties of “NU-BIPHEP(O)s” have not yet been studied. Therefore, we investigated the rotational barriers of **1a** and **3** by enantioselective DHPLC. Elution profiles of **3** were obtained in a temperature range between 50.0 °C and 80.0 °C (CHIRALPAK^®^ IA-3, *t*_ret_ (50 °C) = 4.25 and 8.87 min, α = 2.42, Figure 4A). All rate constants and corresponding Gibbs free energies of activation were directly calculated using the unified equation and the Eyring equation. This elution profile evaluation was done using the DCXplorer software which can be obtained from the corresponding author upon request. A rotational barrier of 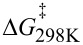 = 99.5 ± 0.1 kJ mol^−1^ was determined for the interconversion of **3**. This is a significant increase compared to unsubstituted BIPHEP(O) (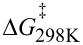 = 88.6 kJ mol^−1^). Eyring plot analysis allowed the determination of the activation parameters Δ*H*^‡^ = 96.3 ± 1.2 kJ mol^−1^ and Δ*S*^‡^ = −10.9 ± 0.2 J (K mol)^−1^ (Figure 4B). The increased rotational barrier of **3** can be rationalized according to additional interactions and steric repulsion of the large 3,5-dichlorobenzoyl amide binding sites. It has to be noted that the entropic contribution to the enantiomerization barrier is exceptionally low.

For comparison, we investigated tetrahydrobiindene “NU-BIPHEP(O)” **1a** in a similar way (see Supporting Information File 1 for details). The elution profiles were evaluated in a temperature range between 20.0 °C and 45.0 °C (CHIRALPAK^®^ IE-3, *t*_ret_ (20 °C) = 14.2 and 26.6 min, α = 1.94). A rotational barrier of 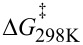 = 92.2 ± 0.3 kJ mol^−1^ was determined and subsequent Eyring plot analysis gave the activation parameters Δ*H*^‡^ = 68.4 ± 1.9 kJ mol^−1^ and Δ*S*^‡^ = −79.9 ± 4.5 J (K mol)^−1^. Interestingly, the activation parameters of **1a** are similar to those reported for 5,5’-dimethoxy BIPHEP(O) (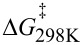 = 93.0 kJ mol^−1^) [20] which is in accordance with a barrier increase caused by small substituents in the 5,5’ positions.

## Conclusion

We report a strategy to use unprotected tetrahydrobiisoindole “NU-BIPHEP(O)” for functionalization with substituents at the secondary amine position, in this study namely by formation of tertiary amide binding sites with 3,5-dichlorobenzoyl chloride. Non-covalent interactions of “NU-BIPHEP(O)s” with Okamoto-type cellulose derivatives resulted in the formation of diastereomeric adducts which led to significant NMR signal splitting in solution and additionally enabled successful on-column deracemization. Furthermore, interconversion barriers of 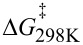 = 92.2 ± 0.3 kJ mol^−1^ and 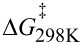 = 99.5 ± 0.1 kJ mol^−1^ were determined by evaluation of enantioselective DHPLC elution profiles quantifying the stereodynamic properties of “NU-BIPHEP(O)” compounds. These results help understanding the influence of substitution patterns on the enantiomerization barrier of BIPHEP ligands and open up new possibilities towards designing tailor-made stereodynamic compounds used as smart ligands in enantioselective catalysis.

## Supporting Information

File 1 Experimental procedures, data for the determination of rotational barriers and copies of NMR spectra.
