# Supplementary material for: Stereodynamic tetrahydrobiisoindole “NU-BIPHEP(O)”s: functionalization, rotational barriers and non-covalent interactions
Source: Beilstein J Org Chem. 2016 Jul 14;12:1453–8. doi: 10.3762/bjoc.12.141 (PMC4979906; doi:10.3762/bjoc.12.141)
Supplement: File 1 — Experimental procedures, data for the determination of rotational barriers and copies of NMR spectra. [file Beilstein_J_Org_Chem-12-1453-s001.pdf]

Supporting Information

for

Stereodynamic tetrahydrobiisoindole "NU-BIPHEP(O)"s: functionalization, rotational barriers and non-covalent interactions

Golo Storch, Sebastian Pallmann, Frank Rominger and Oliver Trapp*

Address: Organisch-Chemisches Institut, Ruprecht-Karls Universität Heidelberg, Im Neuenheimer Feld 270, 69120 Heidelberg, Germany

Email: Oliver Trapp - trapp@oci.uni-heidelberg.de

* Corresponding author

Experimental procedures, data for the determination of rotational barriers and copies of NMR spectra

Table of Contents

1. General remarks S3

  1.1. Synthetic techniques, solvents and chemicals S3

  1.2. NMR Spectroscopy S3

  1.3. Mass spectrometry S3

  1.4. Infrared spectroscopy S4

  1.5. X-ray crystal structure determination S4

  1.6. Chromatography S4

2. Synthetic procedures S5

  2.1. *N*$^{\{Boc\}}$-Tetrahydrobiisoindole “NU-BIPHEP(O)” S5

  2.2. *N*$^{\{H\}}$-Tetrahydrobiisoindole “NU-BIPHEP(O)” S6

  2.3. *N*$^{\{3,5\text{-}DCB\}}$-Tetrahydrobiisoindole “NU-BIPHEP(O)” S7

3. Enantioselective DHPLC S9

  3.1. 6,6’-Bis(diphenylphosphinoyl)-2,3,2’,3’-tetrahydro-1*H*,1*H*’-[5,5’]-biindene S9

  3.2. *N*$^{\{3,5\text{-}DCB\}}$-Biisoindole „NU-BIPHEP(O)“ S10

4. X-ray crystal structure determination of *N*$^{\{Ts\}}$-tetrahydro-biisoindole “Nu-BIPHEP(O)” S11

5. NMR Spectra S12

  5.1. *N*$^{\{Boc\}}$-Tetrahydrobiisoindole “NU-BIPHEP(O)” S12

  5.2. *N*$^{\{H\}}$-Tetrahydrobiisoindole “NU-BIPHEP(O)” S13

  5.3. *N*$^{\{3,5\text{-}DCB\}}$-Tetrahydrobiisoindole “NU-BIPHEP(O)” S15

6. References S16

1. General remarks

1.1. Synthetic techniques, solvents and chemicals

Syntheses with air sensitive reactants were carried out under an argon atmosphere (Ar 5.0) with exclusion of air. All glassware was heated prior to use and standard Schlenk techniques were applied. THF, toluene, diethyl ether, acetonitrile and DCM were dried with an MBraun solvent purification system (MB SPS-800) and stored under argon over molecular sieves. All chemicals were obtained from Sigma-Aldrich, Acros, TCI, abcr or Alfa Aesar and used without further purification. Degassing of solvents was achieved by at least three freeze-pump-thaw cycles.

In order to improve comprehensibility, simplified names were used in some cases rather than using exact IUPAC names. Atom numbering for NMR assignments is not based on IUPAC nomenclature.

1.2. NMR Spectroscopy

NMR spectra were recorded at the NMR Spectroscopy Facility of the Institute of Organic Chemistry (Head: Dr. Jürgen Graf, Heidelberg University) on Bruker Avance 600, 500, 400 and 300 MHz spectrometers. Chemical shifts δ are reported in ppm, coupling constants *J* in Hz and peak multiplicity is defined by s (singlet), d (doublet), t (triplet), q (quartet) and m (multiplet). Broad signals are labeled as such (b/br). The solvent residual signals were used for calibration [1]. Assignment of all signals was realized by two-dimensional experiments (^{1}H-^{1}H-COSY, ^{1}H-^{13}C HSQC-ME and ^{1}H-^{13}C HMBC).

1.3. Mass spectrometry

Mass spectra were recorded at the Mass Spectrometry Facility of the Institute of Organic Chemistry (Head: Dr. Jürgen H. Gross, Heidelberg University) on JEOL JMS-700 Magnetic Sector, Bruker ApexQe hybrid 9.4 T FT-ICR, Finnigan MAT TSQ 700 or EOL AccuTOF GCx time-of-flight spectrometers.

1.4. Infrared spectroscopy

Infrared spectra were recorded on a Thermo Scientific Nicolet 6700 ATR-FT-IR spectrometer.

1.5. X-ray crystal structure determination

Crystal-structure analysis was performed at the X-Ray Crystallography Laboratory of the Institute of Organic Chemistry (Head: Dr. Frank Rominger, Heidelberg University) on Bruker Smart CCD or Bruker APEX diffractometers.

1.6. Chromatography

Thin-layer chromatography was performed with Polygram® Sil G/UV_{254} or Alox N/UV_{254} polyester sheets obtained from Macherey-Nagel. Flash column chromatography was performed using silica (63–200 µm, Sigma-Aldrich) or alumina (neutral, Sigma-Aldrich). HPLC and HPLC-MS measurements were performed on an Agilent Technologies 1200 HPLC equipped with a DAD and a quadrupole mass spectrometer (APCI). All columns with chiral stationary phases were obtained from Chiral Technologies. The HPLC-grade solvents were obtained from Sigma-Aldrich.

2. Synthetic procedures

N-Boc-dipropargylamine [2], 1,4-bis(diphenylphosphinoyl)buta-1,3-diyne [3,4], 6,6′-bis(diphenylphosphinoyl)-2,3,2′,3′-tetrahydro-1*H*,1*H*′-[5,5′]-biindene [3], 6,6′-bis(diphenylphosphinoyl)-2,2′-bis((4-methyl)benzenesulfonyl)-2,3,2′,3′-tetrahydro-1*H*,1*H*′-[5,5′]-biisoindole ($N^{\{Ts\}}$-tetrahydrobiisoindole "Nu-BIPHEP(O)") [3] and cellulose tris(5-fluoro-2-methylphenylcarbamate) [5] are known compounds that were prepared according to the procedures in the given references.

2.1. $N^{\{Boc\}}$-Tetrahydrobiisoindole "NU-BIPHEP(O)"

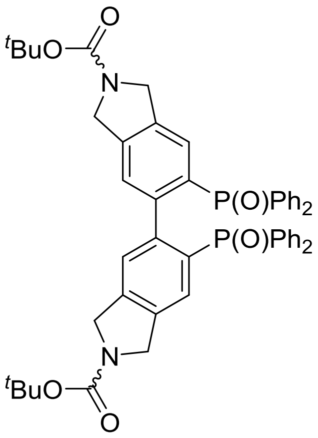

1c

In a first Schlenk flask, $AgBF_4$ (43.2 mg, 222 µmol, 10 mol %) was suspended in degassed anhydrous DCM (4 mL) in the dark. In a second Schlenk flask, [$\{Rh(COD)Cl\}_2$] (54.7 mg, 111 µmol, 5 mol %) was dissolved in degassed anhydrous DCM (4 mL) and BINAP (138 mg, 222 µmol, 10 mol %) was added. In a third Schlenk flask, *N*-boc-dipropargylamine (1.72 g, 8.88 mmol, 4.00 equiv) was dissolved in degassed anhydrous DCM (18 mL). Subsequently, the rhodium complex solution was added to the suspension of $AgBF_4$ followed by 1,4-bis(diphenylphosphinoyl)buta-1,3-diyne (1.00 g, 2.22 mmol, 1.00 equiv). The carbamate solution was added by a syringe pump at a flow rate of 0.7 mL/h by GC capillary at room temperature. After complete addition, the reaction mixture was stirred at room temperature overnight. The reaction mixture was partitioned between EtOAc (100 mL) and brine (100 mL). The organic phase was separated and the aqueous phase was extracted with EtOAc (2 × 50 mL). The combined extracts were dried over Na_2SO_4 and all volatiles were

removed under reduced pressure. The crude product was purified *via* flash column chromatography (silica, hexanes/acetone (5:1) – EtOAc/acetone (5:1) – hexanes/2-propanol (4:1), R_F = 0.70 (hexanes/2-propanol (4:1)).

Beige solid, 1.43 g (77%); The compound exists as mixture of (*E*,*E*), (*E*,*Z*) and (*Z*,*Z*) isomers of the N–C(O) unit. The occurrence of three overlapping signal sets hampers signal assignment in ^{1}H and ^{13}C{^{1}H} NMR; ^{31}P{^{1}H} NMR (CDCl$_3$, 121.64 MHz, 300 K): δ = 29.4 (br); HR-MS (ESI$^+$): m/z calc. for ([M+H]$^+$, [$C_{50}H_{51}N_2O_6P_2$]$^+$): 837.3217, found: 837.3235; IR (FT-ATR): ν (cm^{-1}) = 3054, 2973, 2860, 1691, 1590, 1561, 1474, 1437, 1390, 1365, 1299, 1253, 1167, 1101, 998, 903, 875, 750, 694.

2.2. *N*$^{\{H\}}$-Tetrahydrobiisoindole "NU-BIPHEP(O)"

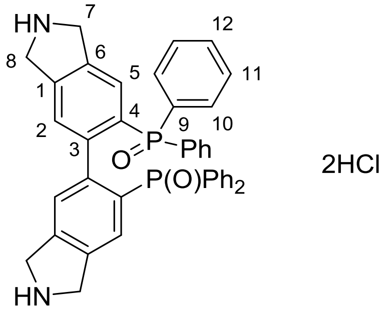

2

1c (200 mg, 239 µmol, 1.00 equiv) was dissolved in anhydrous 2-propanol (4 mL) and a solution of HCl (5–6 M in anhydrous 2-propanol, 2.39 mL, 12.0 mmol, 50.0 equiv) was added. The resulting reaction mixture was stirred overnight. All volatiles were removed under removed pressure. The crude product was re-dissolved in a small amount of 2-propanol and precipitated with EtOAc. The solid was separated and dried under reduced pressure.

Beige solid (**2**·2HCl), 157 mg (93%); ^{1}H NMR (D$_2$O, 600.25 MHz, 300 K): δ = 4.39 (ABq, Δδ = 0.19 ppm, $^2J_{H\text{-}H}$[AB] = 15.4 Hz, 4H, H$^{7/8}$), 4.52 (ABq, Δδ = 0.05 ppm, $^2J_{H\text{-}H}$[AB] = 15.0 Hz, 4H, H$^{7/8}$), 6.80 (d, $^{3/4}J_{H\text{-}P}$ = 3.3 Hz, 2H, H$^{2/5}$), 7.26 (d, $^{3/4}J_{H\text{-}P}$ = 14.0 Hz, 2H, H$^{2/5}$), 7.44-7.69 (m, 20H, H^{10,11,12}), N*H* was not observed; ^{13}C{^{1}H} NMR (D$_2$O, 150.95 MHz, 300 K): δ = 50.4 (2C, C$^{7/8}$), 50.5 (2C, C$^{7/8}$), 126.6 (d, $^{2/3}J_{C\text{-}P}$ = 10.5 Hz, 2C, C$^{2/5}$), 128.3 (d, $^{2/3}J_{C\text{-}P}$ = 13.6 Hz, 2C, C$^{2/5}$),

128.6 (d, $^{2/3}J_{C\text{-}P}$ = 12.5 Hz, 4C, C$^{10/11}$), 128.9 (d, $^{2/3}J_{C\text{-}P}$ = 12.4 Hz, 4C, C$^{10/11}$), 130.3 (d, $^{1}J_{C\text{-}P}$ = 103.6 Hz, 2C, C^{9a}), 130.6 (d, $^{1}J_{C\text{-}P}$ = 104.4 Hz, 2C, C^{4}), 131.0 (d, $^{1}J_{C\text{-}P}$ = 102.2 Hz, 2C, C^{9b}), 131.7 (d, $^{2/3}J_{C\text{-}P}$ = 10.1 Hz, 4C, C$^{10/11}$), 132.0 (d, $^{2/3}J_{C\text{-}P}$ = 10.3 Hz, 4C, C$^{10/11}$), 132.6 (d, $^{4}J_{C\text{-}P}$ = 1.4 Hz, 2C, C^{12a}), 132.8 (d, $^{4}J_{C\text{-}P}$ = 2.1 Hz, 2C, C^{12b}), 134.2 (d, $^{3}J_{C\text{-}P}$ = 14.5 Hz, 2C, C^{6}), 137.8 (d, $^{4}J_{C\text{-}P}$ = 2.0 Hz, 2C, C^{1}), 143.5 (dd, $^{2/3}J_{C\text{-}P}$ = 8.3 Hz, $^{2/3}J_{C\text{-}P}$ = 3.5 Hz, 2C, C^{3}); ^{31}P{^{1}H} NMR (D_2O, 243.00 MHz, 300 K): δ = 34.7; HR-MS (ESI$^+$): m/z calc. for ([M+H]$^+$, [$C_{40}H_{35}N_2O_2P_2$]$^+$): 637.2168, found: 637.2180; IR (FT-ATR, **2**): ν (cm^{-1}) = 3287, 3053, 2848, 1731, 1590, 1466, 1436, 1369, 1242, 1179, 1100, 1071, 1044, 997, 877, 803, 750, 721, 693.

2.3. *N*$^{\{3,5\text{-DCB}\}}$-Tetrahydrobiisoindole "NU-BIPHEP(O)"

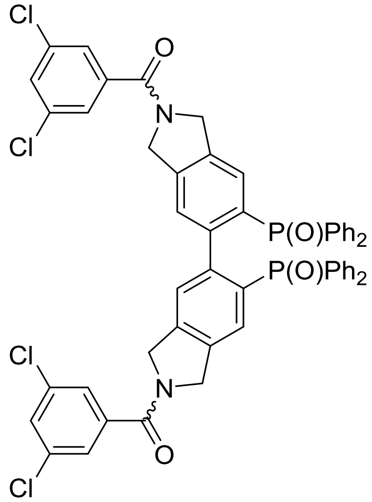

3

2[i] (50.0 mg, 78.5 µmol, 1.00 equiv) was dissolved in degassed anhydrous DCM (2 mL). Anhydrous pyridine (37.3 mg, 471 µmol, 6.00 equiv) and 3,5-dichlorobenzoyl chloride (41.1 mg, 196 µmol, 2.50 equiv) were added and the reaction mixture was stirred overnight at room temperature. The reaction mixture was treated with NH_4Cl solution (saturated aqueous, 20 mL) and DCM (20 mL). The organic phase was separated and the aqueous phase was extracted with DCM (3 × 20 mL). The combined organic extracts were washed with NaOH solution (1 M aqueous, 10 mL) and dried over Na_2SO_4. All volatiles were removed under reduced pressure. The crude product was purified *via* flash column chromatography (silica, EtOAc/acetone (5:1), R_F = 0.27–0.58 (mixture of *E*/*Z* isomers, EtOAc/acetone (5:1))

[i] The hydrogen chloride salt **2·2HCl** is also suitable if additional equivalents of base (e.g. triethylamine) are added.

Reddish solid, 38.0 mg (49%); The compound exists as mixture of (*E*,*E*), (*E*,*Z*) and (*Z*,*Z*) isomers of the tertiary amide unit. The occurrence of three overlapping signal sets hampers signal assignment in ^{1}H and ^{13}C{^{1}H} NMR spectra; ^{31}P{^{1}H} NMR ($CDCl_3$, 243.00 MHz, 300 K): δ = 28.25, 28.30, 28.33, 28.39; HR-MS (ESI^{+}): m/z calc. for ($[M+H]^{+}$, $[C_{54}H_{39}N_2O_4P_2{}^{35}Cl_4]^{+}$): 981.1134, found: 981.1163; IR (FT-ATR): ν (cm^{-1}) = 3056, 2962, 1682, 1634, 1563, 1437, 1395, 1294, 1259, 1181, 1097, 1015, 865, 798, 748, 722, 694.

3. Enantioselective DHPLC

3.1. 6,6'-Bis(diphenylphosphinoyl)-2,3,2',3'-tetrahydro-1*H*,1*H*'-[5,5']-biindene

DHPLC measurements were performed with an CHIRALPAK® IE-3 (150 mm, i.d. 4.6 mm, particle size 3 µm) column (hexane/methanol/2-propanol, 70:15:15, 210 nm, 1.0 mL/min) at temperatures between 20.0 and 45.0 °C.

T [°C]	t_1 [min]	t_2 [min]	h_p [%]	N 1	N 2	α	k_1 [1/s]
20.0	14.187	26.627	0.71	8678	5161	1.94	1.20E-04
20.0	14.179	26.632	0.71	8668	5331	1.94	1.20E-04
20.0	14.155	26.582	0.69	7985	5310	1.94	1.28E-04
25.0	13.345	24.265	0.95	8252	5790	1.88	1.76E-04
25.0	13.359	24.305	1.18	8271	5809	1.89	2.16E-04
25.0	13.354	24.3	1.54	8265	5598	1.89	2.78E-04
30.0	12.612	22.239	2.19	8689	6107	1.83	4.42E-04
30.0	12.607	22.24	2.36	8682	6107	1.83	4.75E-04
30.0	12.599	22.232	1.79	9515	6103	1.83	3.38E-04
35.0	11.927	20.387	2.65	9310	6340	1.77	4.61E-04
35.0	11.9	20.314	2.86	9264	6293	1.77	4.92E-04
35.0	11.9	20.314	3.56	9264	6293	1.77	6.03E-04
40.0	11.272	18.652	5.22	10158	6390	1.72	8.38E-04
40.0	11.279	18.659	5.18	9129	6079	1.72	7.95E-04
40.0	11.287	18.66	5.42	10187	6080	1.72	8.62E-04
45.0	10.737	17.197	9.58	9127	5667	1.66	1.29E-03
45.0	10.747	17.214	9.13	10253	5679	1.66	1.26E-03
45.0	10.745	17.219	10.46	9142	5682	1.66	1.32E-03

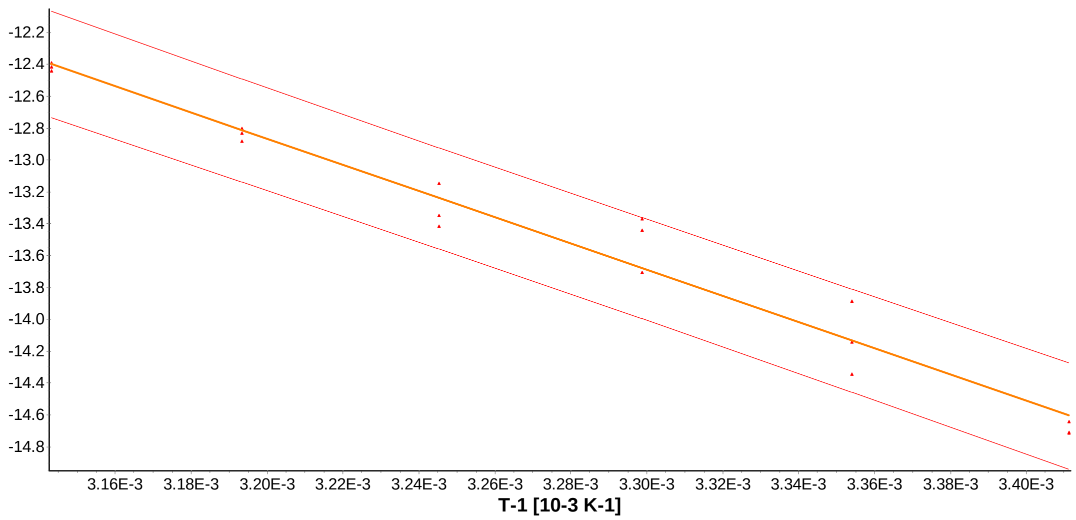

3.2. *N*{3,5-DCB}-Biisoindole „NU-BIPHEP(O)“

DHPLC measurements were performed with an Chiralpak IA-3 (150 mm, i.d. 4.6 mm, particle size 3 µm) column (hexane/2-propanol, 50:50, 210 nm, 1.0 mL/min) at temperatures between 50.0 and 80.0 °C.

T [°C]	t_1 [min]	t_2 [min]	h_p [%]	N 1	N 2	α	k_1 [1/s]
50.0	4.250	8.877	0.57	1464	1777	2.42	2.54E-04
50.0	4.252	8.859	0.56	1466	1769	2.42	2.49E-04
50.0	4.245	8.859	0.51	1460	1769	2.42	2.29E-04
55.0	4.019	8.165	0.94	1682	2042	2.37	4.45E-04
55.0	4.012	8.145	0.90	1674	2031	2.37	4.27E-04
55.0	4.014	8.147	0.86	1677	2032	2.37	4.09E-04
60.0	3.839	7.565	1.54	1746	2334	2.31	7.16E-04
60.0	3.825	7.545	1.50	1729	2320	2.32	6.99E-04
60.0	3.825	7.532	1.55	1729	2129	2.31	7.17E-04
65.0	3.614	6.928	2.85	1761	2485	2.27	1.28E-03
65.0	3.599	6.886	3.00	2107	2233	2.26	1.45E-03
65.0	3.604	6.891	3.20	1748	2455	2.26	1.40E-03
70.0	3.471	6.458	5.26	1904	2574	2.21	2.20E-03
70.0	3.444	6.458	5.14	2300	2574	2.23	2.39E-03
70.0	3.463	6.449	5.14	1892	2858	2.21	2.17E-03
75.0	3.324	6.071	8.95	2080	2775	2.18	3.52E-03
75.0	3.318	6.058	9.20	2069	2761	2.18	3.56E-03
75.0	3.318	6.044	9.26	2069	2746	2.18	3.57E-03
80.0	3.211	5.704	16.79	1882	2696	2.13	5.23E-03
80.0	3.193	5.679	16.89	1852	2667	2.13	5.28E-03
80.0	3.191	5.671	16.87	1849	2658	2.13	5.24E-03

4. X-ray crystal structure determination of *N*{Ts}-tetrahydro-biisoindole “Nu-BIPHEP(O)”

Colourless crystal (plate), dimensions 0.240 × 0.180 × 0.070 mm^3, crystal system orthorhombic, space group Aba2, *Z* = 8, *a* = 15.9120(7) Å, *b* = 28.7697(12) Å, *c* = 22.5183(9) Å, alpha = 90 deg, beta = 90 deg, gamma = 90 deg, *V* = 10308.5(7) $Å^3$, rho = 1.280 g/cm^3, *T* = 200(2) K, $Theta_{max}$ = 23.531 deg, radiation Mo Kalpha, lambda = 0.71073 Å, 0.5 deg omega-scans with CCD area detector, covering the asymmetric unit in reciprocal space with a mean redundancy of 6.86 and a completeness of 99.9% to a resolution of 0.89 Å, 27838 reflections measured, 7633 unique (R(int) = 0.0664), 6120 observed ($I > 2\sigma(I)$), intensities were corrected for Lorentz and polarization effects, an empirical scaling and absorption correction was applied using SADABS [6] based on the Laue symmetry of the reciprocal space, mu = 0.22 mm^{-1}, T_{min} = 0.86, T_{ma} = 0.94, structure refined against F^2 with a Full-matrix least-squares algorithm using the SHELXL-2014/7 (Sheldrick, 2014) software [7], 679 parameters refined, hydrogen atoms were treated using appropriate riding models, except some hydrogens at the disordered solvent methanol, which were not considered at all, Flack absolute structure parameter 0.00(5), goodness of fit 1.02 for observed reflections, final residual values R1(F) = 0.058, wR(F^2) = 0.133 for observed reflections, residual electron density −0.34 to 0.64 $eÅ^{-3}$. CCDC 1473145 contains the supplementary crystallographic data for this paper. These data can be obtained free of charge from The Cambridge Crystallographic Data Centre via www.ccdc.cam.ac.uk/data_request/cif.

5. NMR Spectra

5.1. *N*{Boc}-Tetrahydrobiisoindole "NU-BIPHEP(O)"

$^{31}P\{^{1}H\}$ NMR

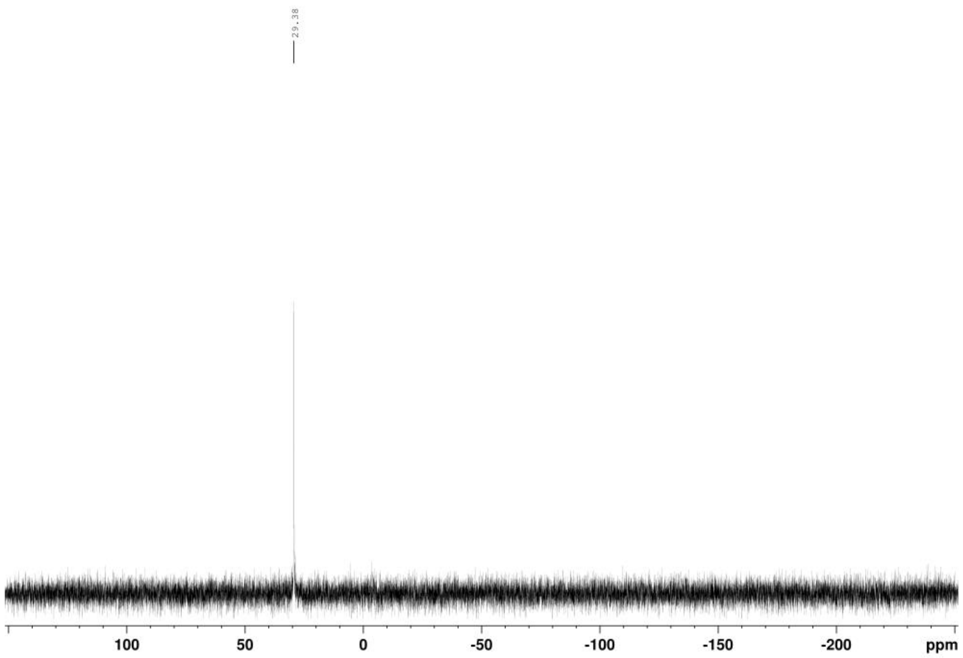

5.2. *N*{H}-Tetrahydrobiisoindole "NU-BIPHEP(O)"

The spectra contain residual 2-propanol that was not separable even when reduced pressure was applied for prolonged time. ^{1}H NMR:

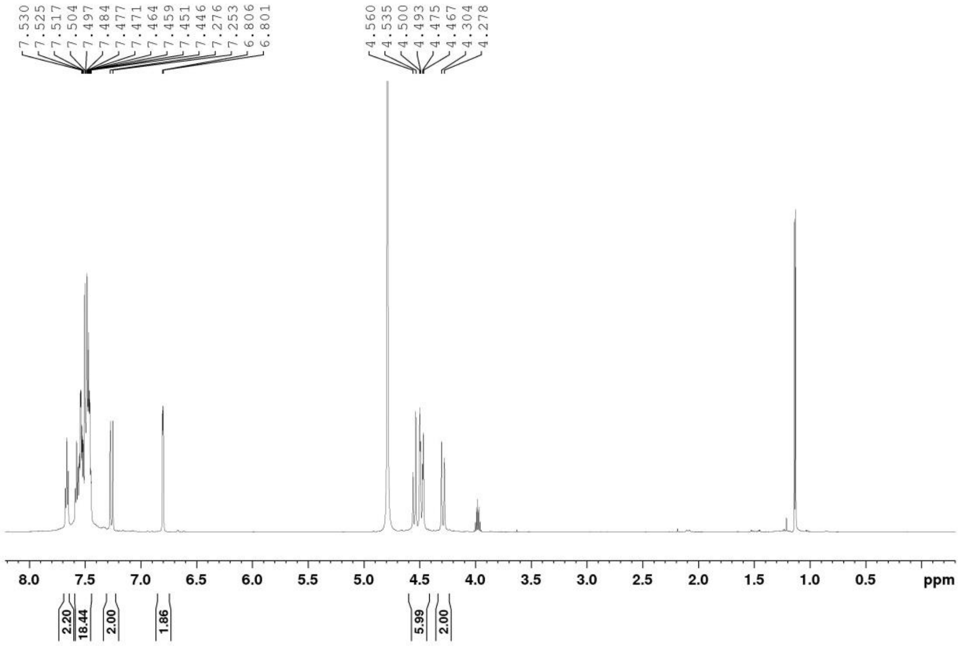

$^{13}C\{^{1}H\}$ NMR

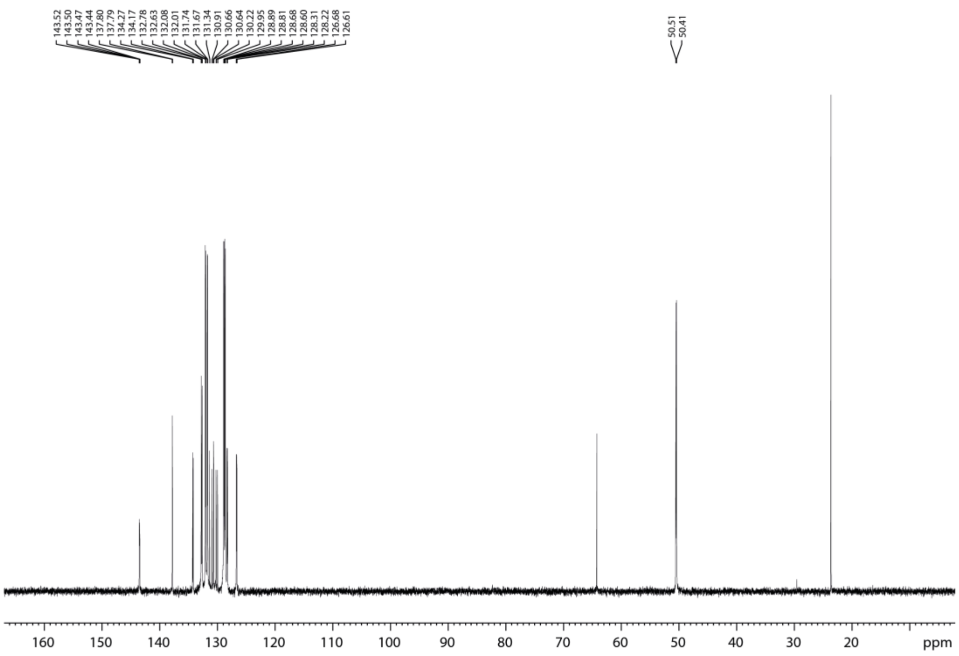

$^{31}P\{^1H\}$ NMR

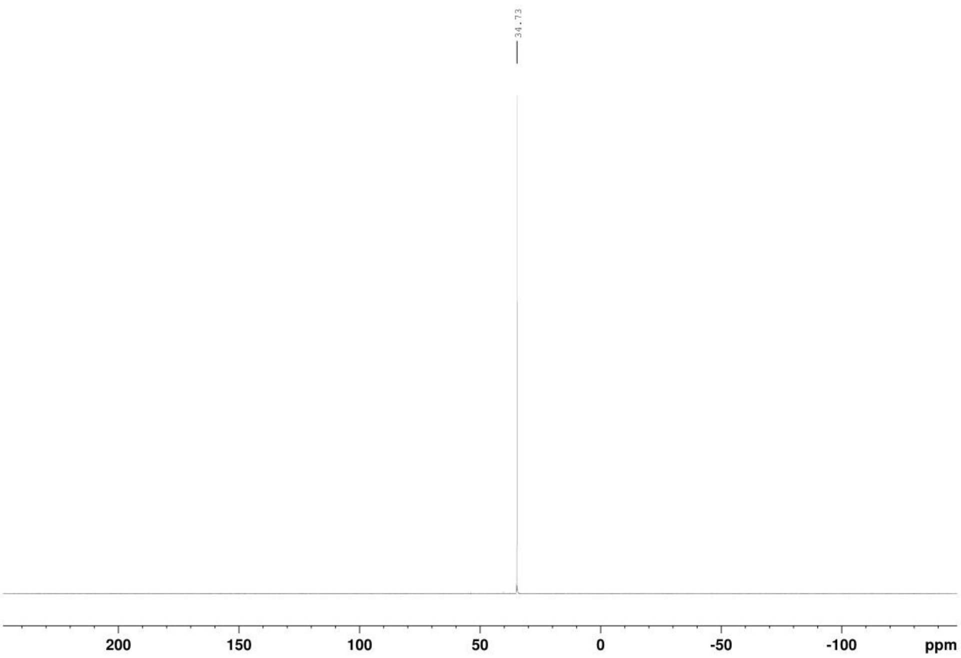

5.3. *N*{3,5-DCB}-Tetrahydrobiisoindole "NU-BIPHEP(O)"

$^{31}P\{^1H\}$ NMR

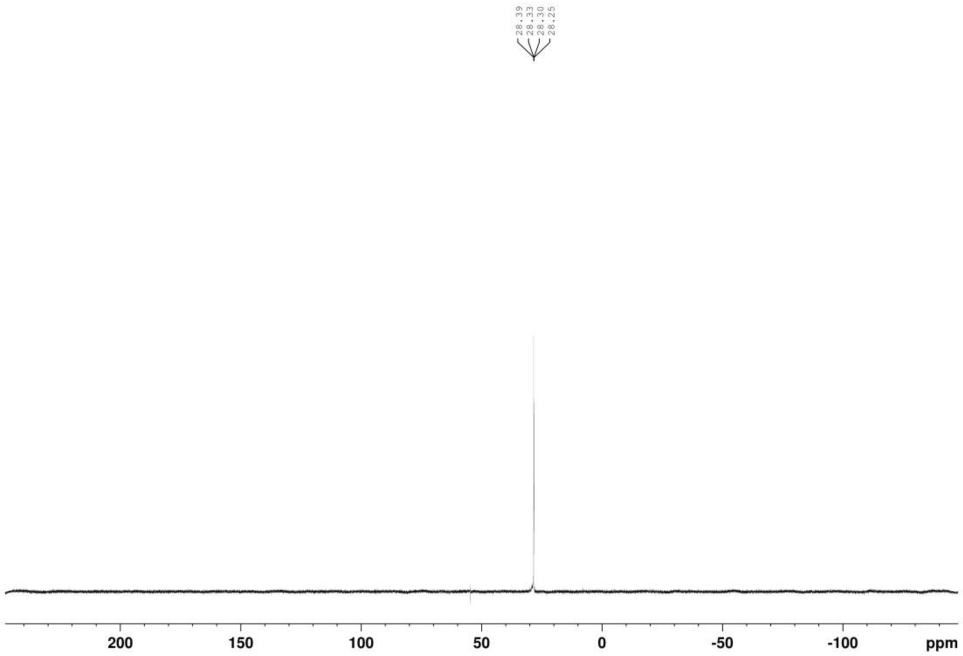

6. References

(1) Fulmer, G. R.; Miller, A. J. M.; Sherden, N. H.; Gottlieb, H. E.; Nudelman, A.; Stoltz, B. M.; Bercaw, J. E.; Goldberg, K. I. *Organometallics* **2010**, *29*, 2176.

(2) Krasia, T. C.; Steinke, J. H. G. *Chem. Commun.* **2002**, *0*, 22.

(3) Doherty, S.; Knight, J. G.; Smyth, C. H.; Harrington, R. W.; Clegg, W. *Org. Lett.* **2007**, *9*, 4925.

(4) Xu, D.; Hong, B. *Angew. Chem. Int. Ed.* **2000**, *39*, 1826.

(5) Yashima, E.; Yamamoto, C.; Okamoto, Y. *J. Am. Chem. Soc.* **1996**, *118*, 4036.

(6) Sheldrick, G. M.; SADABS 2012/1 ed.; Bruker Analytical X-ray-Division: Madison, Wisconsin, 2012.

(7) Sheldrick, G. *Acta Cryst. C* **2015**, *71*, 3.
